# Variation in Proximal Sessile Serrated Lesion Detection Rates During Non-screening Colonoscopies

**DOI:** 10.7759/cureus.82317

**Published:** 2025-04-15

**Authors:** Mo Thoufeeq, Vikram Mohanan, Nilanga Nishad, Eu-wing Toh, Mohamed G Shiha

**Affiliations:** 1 Gastroenterology, Sheffield Teaching Hospitals National Health Service (NHS) Foundation Trust, Sheffield, GBR; 2 Gastroenterology, Kettering General Hospital, Kettering, GBR; 3 Pathology, Sheffield Teaching Hospitals National Health Service (NHS) Foundation Trust, Sheffield, GBR

**Keywords:** colonoscopy surveillance, colorectal cancer, sessile serrated adenoma, sessile serrated lesion, colonoscopy

## Abstract

Aims: Proximal sessile serrated lesions (PSSL) are increasingly recognized as significant precursors of interval colon cancer. We aimed to assess the PSSL detection rates during non-screening colonoscopies and whether there are associations between PSSL detection rate and the established colonoscopy key performance indicators (KPI).

Methods: We retrospectively collected data of all non-screening colonoscopies performed by independent endoscopists at a large teaching hospital between June and December 2019. Data regarding endoscopists’ KPIs, including polyp detection rate (PDR), cecal intubation rate (CIR), and colonoscopy withdrawal time (CWT), were retrieved from the national endoscopy database. SSL resected proximal to the splenic flexure were identified by an expert pathologist. Associations between PSSL detection rate and the different KPIs were assessed using Spearman’s test.

Results: A total of 2956 colonoscopies performed by 33 endoscopists were included. The mean PSSL detection rate was 0.7% (SD 1.5), the mean PDR was 37.1% (SD 17), the mean CIR was 91.3% (SD 6), and the mean CWT was nine minutes (SD 2). There was marked variability in PSSL detection rates between endoscopists (range 0 - 6.5%). PSSL detection rate positively correlated with CWT (r=0.34, p=0.04) but not with the other KPIs.

Conclusion: The wide variability in PSSL detection between endoscopists is concerning of high miss rates and despite achieving the national benchmarks for the established KPIs, many endoscopists still had low PSSL detection rates. Therefore, PSSL detection rate should be considered as an independent KPI.

## Introduction

Colorectal cancer (CRC) is one of the most common malignancies and a major cause of morbidity and mortality worldwide [1]. Colonoscopy and polypectomy of precancerous polyps have been shown to prevent CRC and reduce CRC-related mortality [2]. Unfortunately, colonoscopy does not offer full protection against interval cancer, and some patients develop CRC after an index colonoscopy in which no cancer was found, known as post-colonoscopy colorectal cancer (PCCRC) [3]. Almost 90% of PCCRCs are avoidable and could be attributable to variations in colonoscopy quality between different endoscopists and bowel preparation [4]. Currently, the adenoma detection rate (ADR) has been established as a colonoscopy quality indicator as it is inversely associated with the risk of PCCRC [5]. However, emerging evidence suggests that adenomas are not the sole precursor of CRC and that up to 30% of CRC develops through an alternative serrated pathway [6].

Sessile serrated lesions (SSL) have distinct morphological characteristics compared with conventional adenomas. They are often flat lesions with unclear borders covered with mucous, debris, or bubbles and located in the proximal colon [7]. These features make SSL detection rather challenging for endoscopists, and even when successfully detected, SSL are frequently incompletely resected [8]. This might explain the findings of a recent meta-analysis, which showed a greater prevalence of proximal PCCRC compared with distal PCCRC [9]. Furthermore, a significant proportion of PCCRC shares SSL genetic and molecular features such as microsatellite instability and CpG island methylation [10,11]. A recent study of 277,555 patients with positive fecal immunochemical tests (FIT) undergoing screening colonoscopies found that each 1% increase in the proximal SSL (PSSL) detection rate was associated with a 7% reduction in the risk of PCCRC [12]. Therefore, similar to ADR, efforts should be directed at PSSL detection and removal to reduce the risk of PCCRC.

Despite this increased recognition of PSSL as a significant precursor of CRC, PSSL detection rate is not yet considered a quality metric of colonoscopy. While colonoscopy key quality indicators (KPIs) apply to both screening and non-screening colonoscopies, studies investigating PSSL detection rates have predominantly focused on bowel cancer screening colonoscopies. Thus, we aimed to assess the detection rates of PSSL during non-screening colonoscopies, which constitute the majority of colonoscopy practice, and whether there are associations between the detection rate of PSSL and established colonoscopy KPIs including polyp detection rate (PDR), cecal intubation rate (CIR) and colonoscopy withdrawal time (CWT).

## Materials and methods

Methods

We retrospectively retrieved prospectively collected data of all non-screening colonoscopies performed at Sheffield Teaching Hospitals, United Kingdom, between June and December 2019. The gastroenterology unit is the largest in the UK as it serves a population of more than 1.5 million people and performs nearly 8000 colonoscopies per year.

Non-screening colonoscopies performed by independent endoscopists with at least 30 colonoscopies during the study period were included. Data regarding endoscopists’ KPIs, including PDR, CWT, and CIR, were retrieved from the national endoscopy database (NED) [13].

Proximal lesions were defined as lesions proximal to the splenic flexure (transverse colon, ascending colon, cecum, and ileocecal valve). An expert pathologist identified PSSL according to the updated WHO classification of tumours of the digestive system [14]. Hyperplastic polyps were not included. The PSSL detection rate was defined as the percentage of colonoscopies where one or more PSSL are detected.

Statistical analysis

Statistical analyses were carried out using GraphPad Prism version 9.2.0 for Windows (GraphPad Software, San Diego, CA, USA). Continuous data were expressed as mean (standard deviation), and categorical data were expressed as total numbers (percentages). Endoscopists were divided into two groups: group 1 included those who detected at least one PSSL, and group 2 included those who did not detect any PSSL. Comparisons between the two groups were performed using the unpaired student T-test. Comparisons between the endoscopists according to their specialties were performed using one-way ANOVA with Bonferroni correction for post hoc comparisons. Associations between PSSL detection rate and the different KPIs were assessed using Spearman’s test. Finally, odds ratios were calculated to compare each endoscopist from group 1 to the endoscopist with the highest PSSL detection rate. A two-tailed p-value of <0.05 was considered significant.

## Results

Thirty-three independent endoscopists performed a total of 2,956 non-screening colonoscopies during the study period. The included endoscopists were gastroenterologists (n=21), colorectal surgeons (n=9), and nurse endoscopists (n=3). Overall, the mean PDR was 31.7% (SD 17.1), the mean CWT was 9.0 minutes (SD 2.0), and the mean CIR was 91.3% (SD 6.0).

PSSL was detected by only 12 out of 33 endoscopists (group 1). Table 1 outlines the PSSL detection rates and different KPIs of group 1 endoscopists. The number of colonoscopies with at least one PSSL was 22 (0.7%). There was marked variability in PSSL detection rates between endoscopists (range 0 - 6.5%).

**Table 1:** Group 1 endoscopists PSSL detection rate and KPIs PSSL: proximal sessile serrated lesion, KPI: key performer indicators, PDR: polyp detection rate, CWT: colonoscopy withdrawal time, CIR: cecal intubated rate *Gastroenterologists who are also accredited bowel cancer screening colonoscopists

Endoscopist code	Speciality	Number of colonoscopies	PSSL detection rate (%)	Mean PDR (%)	Mean CWT (min)	Mean CIR (%)
1	Gastroenterologist	31	6.5	19.2	11.1	83.3
2	Gastroenterologist*	169	5.3	63.7	9.1	96.4
3	Gastroenterologist	31	3.2	29.0	8.6	100
4	Gastroenterologist	39	2.6	33.3	8.0	92.3
5	Gastroenterologist*	95	2.1	62.6	9.0	95.2
6	Gastroenterologist	71	1.4	22.9	10.0	85.9
7	Gastroenterologist*	89	1.1	65.4	12.5	95.5
8	Gastroenterologist	103	1.0	24.2	7.8	92.2
9	Nurse endoscopist	122	0.8	25.2	11.0	74.5
10	Colorectal surgeon	166	0.6	26.8	11.9	98.8
11	Gastroenterologist	171	0.6	56.8	9.3	93.5
12	Gastroenterologist	219	0.5	36.6	9.1	90.8

However, we found no differences in PDR, CWT, and CIR between endoscopists who detected PSSL (group 1) and those who did not (group 2), as outlined in Table 2.

**Table 2:** Comparison between endoscopists who detected at least one PSSL (group 1) and those who did not detect any PSSL (group 2) PSSL: proximal sessile serrated lesions, PDR: polyp detection rate, CWT: colonoscopy withdrawal time (in minutes), CIR: cecal intubated rate

	Group 1 (n=12)	Group 2 (n=21)	P value
Number of colonoscopies	1306	1650	0.15
Mean PDR (%)	38.8 (SD 17.9)	27.6 (SD 15.6)	0.06
Mean CWT (min)	9.83 (SD 1.51)	8.56 (SD 2.18)	0.08
Mean CIR (%)	91.6 (SD 7.19)	91.2 (SD 5.34)	0.85

According to endoscopists’ speciality, group 1 had 12 endoscopists with 10 gastroenterologists (83%), one (8.5%) nurse endoscopist, and one (8.5%) colorectal surgeon. Group 2 had 21 endoscopists, a distribution of eight (38%) surgeons, two nurse endoscopists (9.5%), and 11 gastroenterologists (52.5%). There was no statistically significant difference in PDR (p=0.42) between gastroenterologists (mean 34.7%, SD 18.8), nurse endoscopists (mean 27.3%, SD 8.4), and colorectal surgeons (26.2%, SD 14.3). Colorectal surgeons' CWT (mean 7.35 min, SD 0.8) was significantly shorter than gastroenterologists (mean 9.40 min, SD 1.9) and nurse endoscopists (mean 11.4 min, SD 0.9) (p=0.001). Nurse endoscopists had lower CIR (mean 79.5%, SD 4.2) compared with gastroenterologists (93%, SD 5.1) and colorectal surgeons (91.5%, SD 3.4) (p=0.0003). The three groups had similar PSSL detection rates (p=0.39): gastroenterologists (mean 1.0%, SD 1.8), colorectal surgeons (mean 0.28%, SD 0.8), and nurse endoscopists (mean 0.26%, SD 0.4).

**Figure 1:**
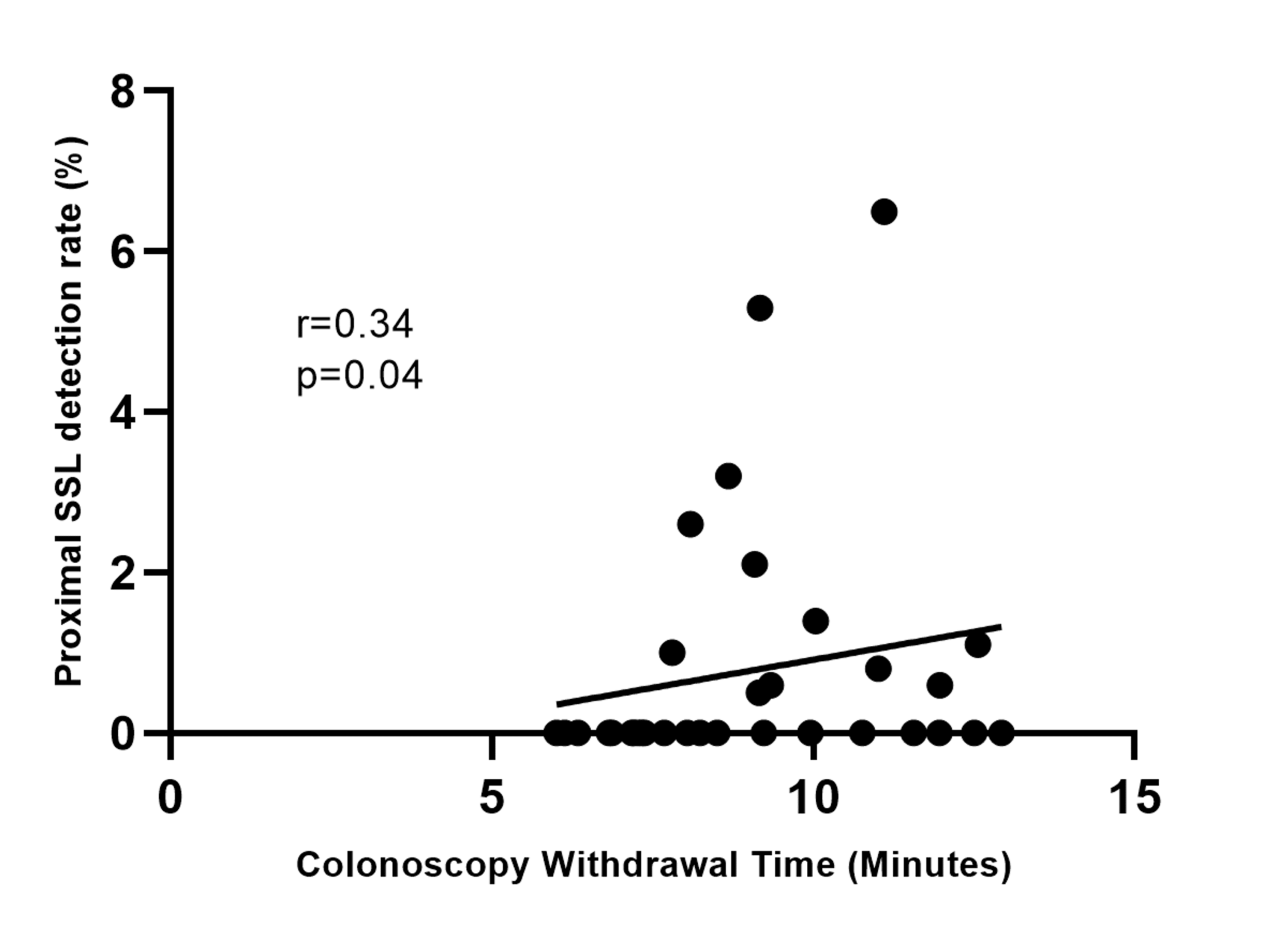
Correlation between colonoscopy withdrawal time and PSSL detection rate PSSL: proximal sessile serrated lesions

There was only a weak positive correlation between PSSL detection rate and colonoscopy withdrawal time (r=0.34; p=0.04), as shown in Figure 1. We found no association between PSSL detection rate and total number of colonoscopies performed by endoscopists (r=0.12; p=0.50), PDR (r=0.31; p=0.07), and CIR (r=0.09; p=058).

The odds for detection of at least one PSSL for individual group 1 endoscopists compared with endoscopist 1 (highest level detector) ranged from 0.06 to 0.81 (Table 3). 

**Table 3:** Odds for detection of at least one PSSL for individual group 1 endoscopists compared with endoscopist 1 (highest level detector) PSSL: proximal sessile serrated lesions

Endoscopist	Odds ratio (95% CI)	P value
Endoscopist 2	0.81 (0.19 – 3.92)	0.68
Endoscopist 3	0.48 (0.03 – 4.37)	0.99
Endoscopist 4	0.38 (0.02 – 3.44)	0.58
Endoscopist 5	0.31 (0.04 – 2.08)	0.25
Endoscopist 6	0.20 (0.01 – 1.86)	0.21
Endoscopist 7	0.16 (0.01 – 1.48)	0.16
Endoscopist 8	0.14 (0.00 – 1.27)	0.13
Endoscopist 9	0.11 (0.00 – 1.07)	0.10
Endoscopist 10	0.08 (0.00 – 0.79)	0.06
Endoscopist 11	0.08 (0.00 – 0.76)	0.06
Endoscopist 12	0.06 (0.00 – 0.59)	0.04

## Discussion

In this retrospective study of 2956 non-screening colonoscopies, the overall PSSL detection rate was 0.7%. We found wide variations in PSSL detection between different endoscopists, which is concerning that many lesions may have been missed. Detection of PSSL was associated with longer colonoscopy withdrawal time but not with other colonoscopy quality indicators.

A recent meta-analysis of average-risk screening colonoscopies reported a pooled PSSL detection rate of 1.6% (95% CI 1.1 - 2.4%) [15]. A higher PSSL detection rate of 5.6% was reported in patients with positive FIT undergoing screening colonoscopies [16]. We found that in low-risk patients undergoing non-screening colonoscopies, the PSSL detection rate is 0.7%. This contrast in PSSL detection rates between the different studies is likely attributable to the different patient populations and risk for CRC.

The wide variation of PSSL detection between endoscopists aligns with the published literature. The inter-endoscopist variability in detecting proximal polyps is significantly higher than distal polyps and twofold higher for serrated lesions than adenomas [17]. Moreover, PSSL detection rates vary greatly between different centres; a cohort study of 32 endoscopy centres reported a PSSL detection rate range of 0% to 9.8% [18]. Similar to ADR, gastroenterologists have a higher PSSL detection rate than surgeons [16]. While we did not find a significant difference in PSSL detection rate between gastroenterologists, surgeons, and nurse endoscopists, surgeons had a shorter CWT, which will affect their PSSL detection rate with higher colonoscopy volume. These findings indicate that the miss rates of precancerous PSSL are significant and potentially contribute to a large proportion of interval cancers. Indeed, a recent study demonstrated a significantly higher risk of PCCRC after index colonoscopy performed by endoscopists with an SSL detection rate of <3% [19]. Another retrospective cohort study of 229,729 people undergoing screening colonoscopies in Austria showed that a rise in PSSL detection rate was associated with a significant reduction in the risk of PCCRC death (HR 97; 95% CI, 0.94 - 0.99; p=0.02) [20].

Despite the high PDR in our study, which exceeds the national aspirational benchmark of 20%, the PSSL detection rate was low and was not associated with PDR [21]. Studies examining the association between SSL detection rate and PDR have been conflicting [18, 22-24]. These findings indicate that endoscopists with high PDR may not always achieve an adequate PSSL detection rate, limiting the utility of using ADR or PDR as a surrogate for SSL detection. 

In the current study, we found a weak correlation between the PSSL detection rate and longer colonoscopy withdrawal time but not with PDR or CIR. This accords with the findings of De Wijkerslooth et al., who showed that colonoscopy withdrawal time was the only factor associated with PSSL detection in a prospective study of 1354 patients undergoing screening colonoscopies [25]. Although a colonoscopy withdrawal time of at least six minutes is required to achieve an adequate ADR, a longer colonoscopy withdrawal time is required to detect PSSL. Patel et al. demonstrated that endoscopists with a mean colonoscopy withdrawal time of 11 minutes are twice as likely to detect PSSL compared with those with shorter withdrawal times [26]. Interestingly, we did not find a statistically significant difference in mean colonoscopy withdrawal time between endoscopists who detected at least one PSSL and those who did not. These findings suggest that achieving the current KPIs in colonoscopy does not necessarily lead to an increased PSSL detection rate. 

Amid the growing evidence of the wide variability in PSSL detection between endoscopists and the malignant potential of these lesions, the PSSL detection rate has been proposed as a quality metric in colonoscopy to reduce the risk of interval cancer. However, unlike conventional adenomas, there is no agreed benchmark for PSSL detection rates as they vary greatly between different studies according to the study population and the SSL definition used [15]. Moreover, there is significant inter-pathologist variability in reporting SSL [27]. In fact, pathologists in some centres may never report serrated lesions [18]. While these factors hinder the use of PSSL as a quality metric in colonoscopy at present, future studies should focus on determining targets for PSSL detection rate, variables that influence PSSL detection, and whether focused training and quality improvement interventions can increase detection rates for both experienced endoscopists and trainees.

The strengths of this study include the use of a clearly defined classification of serrated lesions by an expert pathologist and the relatively large number of colonoscopies and endoscopists included. However, the current study has several limitations. First, the retrospective design of the study may have failed to account for potential confounders, particularly colonoscopy indications and patient factors. Nonetheless, studies have consistently shown that serrated lesion detection is predominantly influenced by the endoscopist’s characteristics rather than patient or procedure-associated factors [16,28,29]. Second, it has been suggested that a minimum of 500 colonoscopies are required to assess an endoscopist’s PSSL detection rate [24]. Hence, we may have underestimated the true PSSL detection rate for the individual endoscopists included in the study. However, this would not explain the wide inter-endoscopist variation in PSSL detection that we observed. Third, as the PSSL detection rate included in the current study was based only on the pathological results, we may have missed PSSL that were not resected during the study period. Finally, as this is a single-center study, the results may not be generalizable.

**Figure 2:**
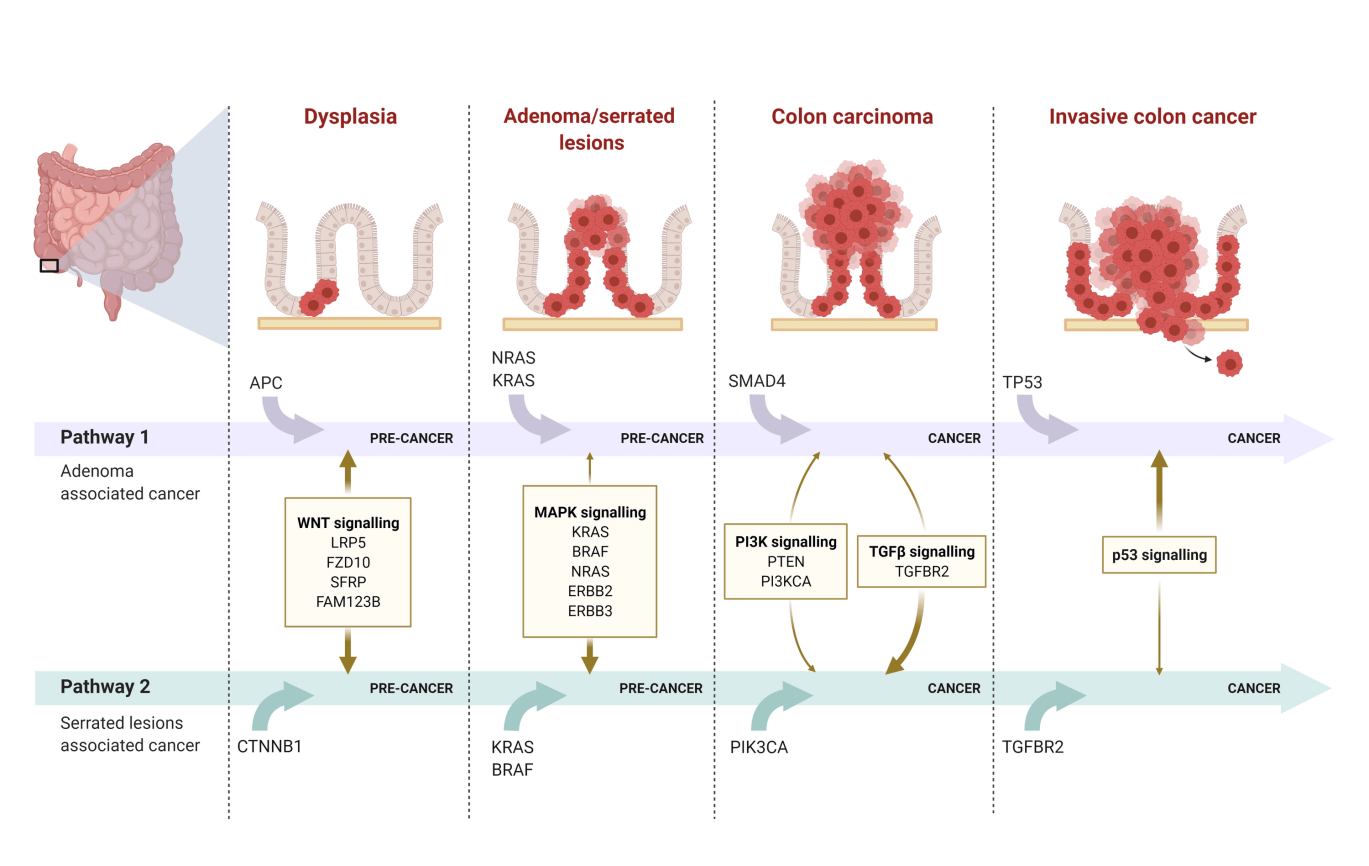
Colorectal cancer arises from either the conventional adenoma-carcinoma pathway (Pathway 1) or the alternative serrated pathway (Pathway 2); cancers arising from both pathways exhibit distinct molecular alterations The figure is adapted from “Colon cancer progression,” by BioRender.com (2022); retrieved from https://app.biorender.com/biorender-templates

These are particularly important as CRC also develops through an alternative serrated pathway (Figure 2) and not only via the conventional adenoma-carcinoma pathway.

The advent of artificial intelligence and computer-aided detection of polyps (CADe) may contribute to increased PSSL detection rates. Using CADe during colonoscopy could help overcome the limitations of human perception when it comes to detecting subtle and flat lesions. A recent systematic review and meta-analysis found that using CADe increased the detection of adenomas (RR 1.26; 95% CI, 1.18 - 1.35) and polyps (RR 1.32; 95% CI, 1.19 - 1.47) compared with standard colonoscopy. However, the impact of CADe on SSL detection rates was less conclusive (RR 1.14; 95% CI, 0.84 - 1.56) [30]. This highlights the need for more studies with larger training datasets focusing on SSL detection rates as their primary end point.

## Conclusions

In conclusion, there is a wide variability in PSSL detection rates between endoscopists, which is concerning of high miss rates, and despite achieving the national benchmarks for the established KPIs, many endoscopists still had low PSSL detection rates. Therefore, PSSL detection rate should be considered as an independent KPI in colonoscopy.
